# Induction of Remission in Pediatric Crohn’s Disease Patients Assessed by the Mucosal Inflammation Noninvasive Index

**DOI:** 10.3390/jcm10235613

**Published:** 2021-11-29

**Authors:** Roma Herman, Paulina Dumnicka, Stanisław Pieczarkowski, Krzysztof Fyderek

**Affiliations:** 1 Department of Pediatrics, Gastroenterology and Nutrition, University Children’s Hospital of Cracow, Jagiellonian University Medical College, Wielicka 265, 30-663 Kraków, Poland; stpiecz@wp.pl (S.P.); kfyderek@usdk.pl (K.F.); 2 Department of Medical Diagnostics, Faculty of Pharmacy, Jagiellonian University Medical College, Medyczna 9, 30-688 Kraków, Poland; paulina.dumnicka@uj.edu.pl

**Keywords:** pediatrics, Crohn’s disease, exclusive enteral nutrition, fecal calprotectin

## Abstract

Mucosal healing (MH) is the main therapeutic goal of Crohn’s disease (CD). The Mucosal Inflammation Noninvasive Index (MINI) appears to be a promising tool for distinguishing MH from its inflammation. This study aims to evaluate MINI in monitoring remissions induced by exclusive enteral nutrition (EEN) in pediatric CD patients. Out of 55 newly diagnosed CD children, 31 who completed 6–8 weeks of EEN were analyzed. Clinical and biochemical data, activity of CD assessed with the Pediatric Crohn’s Disease Activity Index (PCDAI) and MINI were compared within seven days pre- and post-EEN. Response to induction therapy was defined as a decrease of PCDAI by >12.5 points. The follow-up was performed up to 12 months after EEN termination. Out of 31 children who completed 6–8 weeks of EEN, eight required corticosteroids in addition to EEN. Twenty-four patients (77%) responded to induction therapy. In responders, MINI decreased from 19 (Q1:17; Q3:22) to 12 (Q1:6; Q3:14), *p* < 0.001. The diagnostic accuracy of post-EEN MINI and post-EEN fecal calprotectin (FC) for treatment failure were AUC: 0.899 (95%CI: 0.737–1.000) and 0.762 (95%CI: 0.570–0.954), respectively. In the follow-up of 25 patients (80.6%), the post-EEN MINI of ≥13 points predicted CD relapse (87.5% sensitivity; 64.7% specificity), while FC had no prognostic value. MINI allows for monitoring of EEN and is superior in predicting disease relapse to FC.

## 1. Introduction

In children with active luminal Crohn’s disease (CD), therapy with exclusive enteral nutrition (EEN) is recommended as the first line for induction of remission [1]. EEN is defined as feeding with complete liquid formula as the sole source of food [1]. Early complete mucosal healing (MH) after EEN induction in children predicts sustained remission [2]. If EEN is insufficiently effective after 2–4 weeks of good compliance, systemic corticosteroids (CS) may be considered for inducing remission [1]. There are no evidence-based guidelines of when best to re-evaluate disease activity after initiation of induction therapy. The use of endoscopic evaluation to assess MH after induction of remission in the pediatric population is impractical and has several limitations. Since clinical scores alone, such as Pediatric Crohn’s Disease Activity Index (PCDAI) or the weighted PCDAI (wPCDAI) [3], do not adequately reflect mucosal healing, fecal calprotectin (FC) is used as a superior measure of mucosal inflammation despite the fact that treatment modification based solely on fecal calprotectin is not recommended [1,3]. The recently published Mucosal Inflammation Noninvasive index (MINI) [4] is a simple and intuitive clinimetric tool developed to discriminate MH from mucosal inflammation. The authors of MINI revealed that the index was significantly more accurate than FC (*p* = 0.013) in assessing MH, which can be relevant especially in children with lower ranges of FC (100–599 µg/g) [4]. This study aims to assess the usefulness of MINI in evaluating the clinical response to induction of remission started with EEN in the pediatric population. We hypothesized that MINI allows for simple, noninvasive and adequate estimation of the effectiveness of induction treatment in children with CD.

## 2. Materials and Methods

### 2.1. Study Design

This preliminary observational, single-center study involved consecutive newly diagnosed CD pediatric patients (diagnosed between March 2015 and September 2016 at the Department of Pediatric Gastroenterology and Nutrition, University Children’s Hospital of Cracow, Poland). Patients who had incomplete medical records, unsuccessful ileo-cecal valve intubation, concomitant biological treatment, contraindications to EEN, isolated perianal disease, penetrating phenotype of the disease or those who did not complete six weeks of EEN were excluded. The collection of biochemical, clinical data and PCDAI calculation was performed within seven days before induction of EEN (pre-EEN) and repeated within seven days after EEN completion (post-EEN). Standard induction treatment with EEN was performed with polymeric feeding, individually selected for each patient by the multidisciplinary Clinical Nutrition Team. The EEN was administered for at least six weeks, after which maintenance enteral nutrition (MEN) or normal diet was gradually reintroduced. The diet in 30 children was administered via nasogastric tube and, in one patient, orally. If EEN was insufficiently effective, the treatment with CS (methylprednisolone or budesonide) was co-administered to EEN. The patients with perianal disease were treated according to commonly accepted criteria [5]. If patients with perianal disease did not respond to conventional treatment, they were qualified for biological treatment and excluded from the study. Concomitant therapy with early thiopurines started within three months from the diagnosis. It was administered in 29 patients (azathioprine 2–2.5 mg/kg/day or 6-mercaptopurine 1–1.5 mg/kg/day). Data on the number of CD exacerbations, defined as the need for hospitalization due to worsening of CD symptoms, were collected from children completing one year of follow-ups.

The primary objective of the study was to compare the MINI pre- and post-induction therapy in children who achieved and those who did not achieve clinical response. The secondary objective involved finding associations between post-EEN MINI score, post-EEN FC and the number of exacerbations during one year of follow-up. Additionally, we aimed to assess FC pre- and post-EEN.

### 2.2. Reference Standards

The diagnosis was made according to accepted diagnostic criteria for pediatric CD [6]. The location and clinical manifestation of the disease were evaluated according to the Paris classification [7]. Perianal disease was defined as the presence of fistula, abscess, fissure, associated uncomplicated skin inflammation or skin tags. Disease activity was scored using the PCDAI in the following way: remission <10 points, mild 10–27.5 points, moderate 30–37.5 points, severe 37.5–100 points [8]. Clinical response was defined as a decrease of the PCDAI score of >12.5 points. Phenotype data, including patient characteristics, PCDAI, biochemistry and FC, were collected prospectively for the first time within seven days before induction of EEN (pre-EEN). The second evaluation of patient characteristics, disease activity, biochemistry and FC was done within seven days after completing EEN (post-EEN). The fecal sample was collected in the hospital and was transferred to the laboratory in a plastic container on the day of collection. The samples were tested using the immunochromatographic point-of-care test (Bühlmann Quantum Blue^®^ fCAL assay, Bühlmann Laboratories AG, Schönenbuch, Switzerland) at two levels (LF-CAL25, 30–300 µg/g and LF-CHR25 100–1800 μg/g).

The MINI index evaluates the following main categories: stool pattern, FC, erythrocyte sedimentation rate (ESR) and C-reactive protein (CRP). The calculation of MINI and the interpretation of the total score was performed according to recommendations by Martinus A. Cozijnsen et al. [4]. The maximal score of MINI is 25 points and minimal minus 3 points. The interpretation of MINI was made in the following way: <8 MINI points reflected MH, 8–11 MINI points characterized mild inflammation and >11 MINI points indicated moderate inflammation [4]. In this study, the MINI index was calculated retrospectively on the basis of prospectively collected data on stool patterns and inflammatory markers.

### 2.3. Statistical Analysis

The number of patients and percentage of the appropriate group were reported for categories. Contingency tables were analyzed with the chi-squared or Fisher exact tests. Mean and standard deviation (SD) or median, lower quartile (Q1) and upper quartile (Q3) were reported for quantitative variables with or without a normal distribution (assessed with Kolmogorov-Smirnov test), respectively. Data were compared between induction therapy responders and non-responders using a *t*-test or Mann-Whitney test, according to the variables’ distribution. Pre-EEN and post-EEN values were compared with the *t*-test for dependent samples or Wilcoxon matched pairs test, respectively. The Spearman rank-order correlation coefficient was applied to assess correlations of FC and MINI (non-normally distributed variables). Receiver operating characteristic (ROC) curves were used to assess and compare the diagnostic accuracy of studied variables for induction treatment failure. The cut-off values were selected at the maximum Youden index. For comparison, we also provided information about the cut-off, enabling 100% sensitivity for induction treatment failure. Multiple logistic regression was used to assess whether post-EEN MINI was associated with induction treatment outcome independently of steroid treatment: post-EEN MINI and steroid use were included as independent variables in the model. The statistical tests were two-tailed; *p* < 0.05 indicated statistical significance. Statistica 13.3 (Tibco Inc., Tulsa, OK, USA) and dedicated medical bundle 4.0 (StatSoft Poland, Kraków, Poland) software were used for computation.

### 2.4. Ethical Considerations

This single-institution study was approved by the Bioethics Committee of the Jagiellonian University, Kraków (No: 122.6120.52.2015). Signed informed consent was obtained from all families before enrolment.

## 3. Results

### 3.1. Baseline Characteristics of Studied Patients

**Figure 1:**
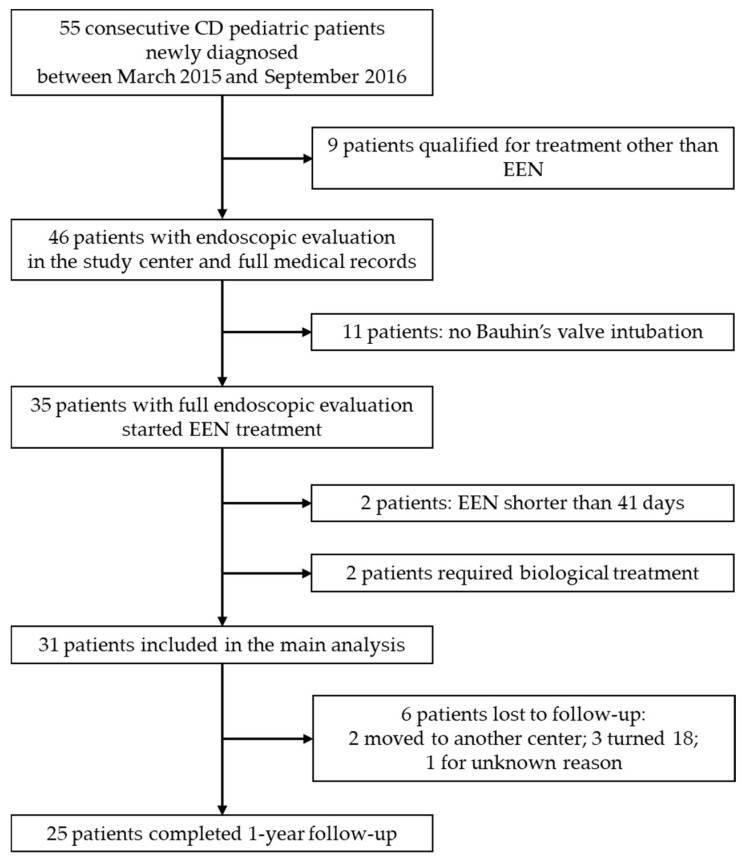
Flow diagram of patients’ selection. Abbreviations: CD, Crohn’s disease; EEN, exclusive enteral nutrition.

The study involved 55 consecutive newly diagnosed CD pediatric patients. From this group, 46 children were qualified for exclusive enteral nutrition (EEN) as the first line for induction of remission. Eventually, 31 eligible children, 19 (61.3%) males, at the mean age of 12 ± 3.9 years, were included in the study (Figure 1). The baseline characteristic of the group, and its phenotype according to the Paris Classification, is shown in Table 1. Patients with perianal manifestation had concomitant intestinal lesions. In all patients with perianal manifestation, conventional treatment was successful. The patient with B2B3 phenotype was initially diagnosed with stricturing disease, and the modification of the Paris classification was performed after receiving the magnetic resonance enterography (MRE) result, post-EEN. Steroid treatment due to insufficient effects of EEN was co-administered in eight patients (25.8%).

Before introducing EEN, patients in our cohort had moderate to severe activity of the disease with pre-EEN median PCDAI and MINI scores of 32 (Q1:20; Q3:38) and 20 (Q1:15; Q3:22) points, respectively. Pre-EEN median FC was 1800 µg/g (Q1:1053; Q3:1800). There were no significant associations between MINI or FC and disease phenotype (localization, growth retardation and disease behavior). Moderate correlation between pre-EEN MINI and pre-EEN PCDAI (*R* = 0.39; *p* = 0.029) was observed. In contrast, we did not find the correlation between pre-EEN FC and pre-EEN PCDAI (*R* = 0.23; *p* = 0.2).

### 3.2. The Associations between Clinical Scores, Laboratory Tests and EEN Treatment Results

**Figure 2:**
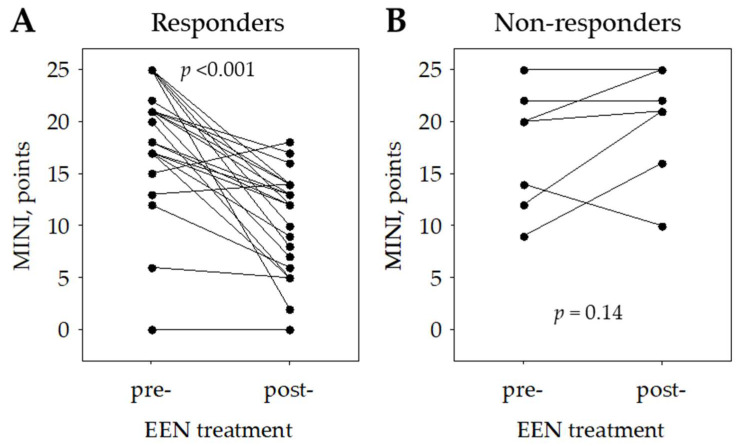
MINI score in responders (**A**) and non-responders (**B**) to induction of remission. Abbreviations: EEN, exclusive enteral nutrition; MINI, Mucosal Inflammation Noninvasive Index.

The patients underwent 6–8 weeks of EEN treatment. The mean duration of EEN was 53 ± 9 days. Five patients (16.1%) were additionally administered methylprednisolone, and three patients (9.7%) budesonide. In total, 24 children (77%) achieved clinical response, and in those patients, we observed a significant decrease in the median MINI score from 19 (Q1: 17; Q3: 22) to 12 (Q1: 6; Q3: 14) points; *p* < 0.001. The median MINI score increased in non-responders from 20 (Q1: 12; Q3: 22) to 21 (Q1: 16; Q3: 25) points; *p* = 0.14 (Figure 2).

In responders, FC concentration after EEN decreased by median of 258 µg/g (Q1:0; Q3:1414). For non-responders median pre- minus post-EEN difference in FC concentration was zero (Q1: −1413; Q3: 213); *p* = 0.019 (Table 2).

**Figure 3:**
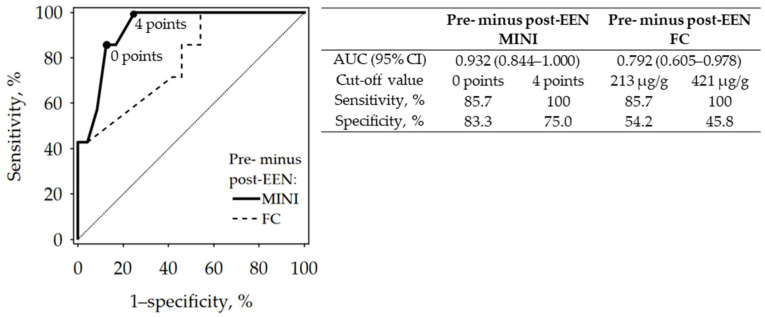
ROC curves showing the diagnostic accuracy of pre- minus post-EEN MINI and FC for the diagnosis of induction treatment failure (black points indicate the selected cut-off values of pre- minus post-EEN difference in MINI). Abbreviations: AUC, area under the ROC curve; CI, confidence interval; EEN, exclusive enteral nutrition; FC, fecal calprotectin; MINI, Mucosal Inflammation Noninvasive Index.

Reduction of MINI by fewer than four points after EEN treatment was prognostic for its failure (Figure 3).

**Figure 4:**
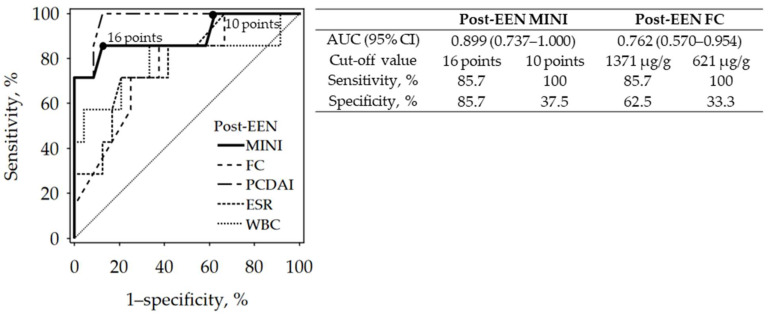
ROC curves showing the diagnostic accuracy of post-EEN MINI (as compared to other post-EEN data) for the diagnosis of induction treatment failure (black points indicate the cut-off values of post-EEN MINI). Abbreviations: AUC, area under the ROC curve; CI, confidence interval; EEN, exclusive enteral nutrition; ESR, erythrocyte sedimentation rate; FC, fecal calprotectin; MINI, Mucosal Inflammation Noninvasive Index; PCDAI, Pediatric Crohn’s Disease Activity Index; WBC, white blood cell count.

Further analysis revealed that the diagnostic accuracy of post-EEN MINI for treatment failure was high. The comparison of diagnostic performance of other post-treatment laboratory tests is shown in Figure 4. The areas under the ROC curves (AUC) of post-EEN MINI and FC did not differ significantly, as reflected in the overlapping 95% confidence intervals for AUC. Additionally, the post-treatment correlation between MINI and PCDAI (*R* = 0.73; *p* < 0.001) was stronger than between FC and PCDAI (*R* = 0.59; *p* < 0.001).

Steroid treatment was required in four patients (16.7%) who achieved remission and four (57.1%) who did not (*p* = 0.031). Only post-treatment WBC (median 11.5 vs. 6.9 × 10^3^/µL; *p* = 0.002) and MINI (median 15.5 vs. 13.0 points; *p* = 0.049) differed significantly in patients who received CS and those who did not. In logistic regression analysis, post-treatment MINI predicted induction treatment failure independently of CS treatment (odds ratio 1.57 per 1-point increase in MINI; 95% confidence interval 1.08–2.28; *p* = 0.014).

### 3.3. The Associations between Clinical Scores, Laboratory Tests and CD Course during 1-Year Follow-Up Post EEN

**Figure 5:**
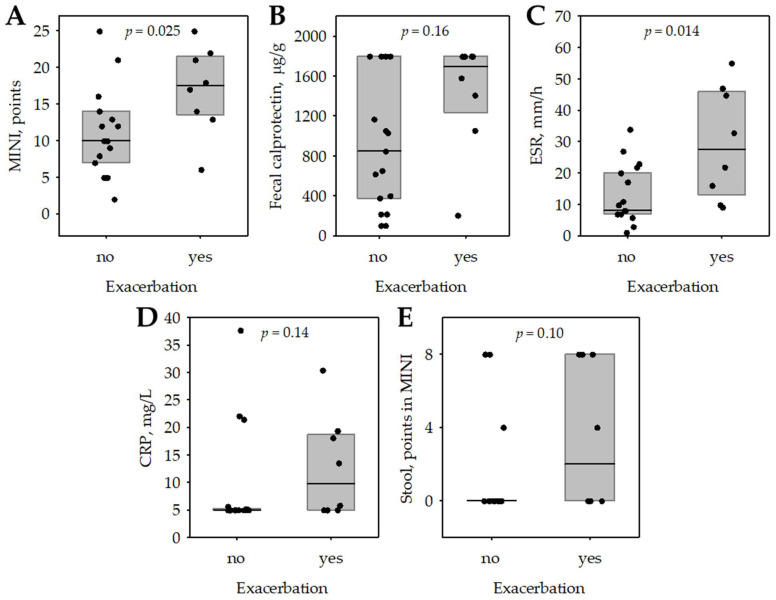
The association between post-EEN MINI score (**A**) and the items included in MINI: post-EEN fecal calprotectin (**B**), erythrocyte sedimentation rate (**C**), C-reactive protein (**D**), stool pattern (**E**) and the incidence of Crohn’s disease exacerbation during 1-year follow-up after finishing EEN treatment. Median (central line); interquartile range (box); raw data (points); *p*-values were calculated in Mann-Whitney test. Abbreviations: CRP, C-reactive protein; ESR, erythrocyte sedimentation rate; MINI, Mucosal Inflammation Noninvasive Index.

Out of 31 children who completed 6–8 weeks of EEN, one-year follow-up data were available for 25 patients (80.6%). Out of the other six patients, three children changed treatment centers, two turned 18 and in one patient, the reason for loss to follow-up was unknown. In the follow-up group, eight children required hospital treatment due to exacerbation. Their median post-EEN MINI score was higher than that of the remaining 17 patients (17.5 (Q1: 13.5; Q3:21.5) points vs 10.0 (Q1:7.0; Q3:14.0) points; *p* = 0.026). FC did not differ between the group of children who experienced relapse and the one with sustained response to induction therapy (*p* = 0.16) (Figure 5). Post-EEN MINI predicted CD exacerbations during one-year follow-up with a diagnostic sensitivity of 87.5% and specificity of 64.7% at the cut-off of 13 points; the area under the ROC curve was 0.783 (95% confidence interval: 0.583–0.983).

## 4. Discussion

The last decade brought a shift in therapeutic goals in pediatric CD, from symptom control to mucosal and transmural healing. What is more, complete MH after EEN induction predicts sustained remission [2]. The gold standard for assessing MH is ileocolonoscopy, which is invasive and cumbersome in pediatric patients. Therefore, surrogate markers of MH are investigated. Studies in adult and pediatric CD patients have shown that calprotectin correlates well with endoscopic scores [9]. However, its large interpatient variability prevents determining a clear cut-off value to reflect MH [10]. According to updated ECCO-ESPGHAN guidelines, in a patient following induction therapy, a decrease in FC in the context of clinical improvement can be used as a marker of treatment response [1]. The MINI index was developed to combine subjective but relevant clinical symptoms with objective measurements such as serum and fecal inflammatory markers to adequately discriminate MH from mucosal inflammation in pediatric CD patients [4]. The adaptation and validation of MINI for Crohn’s disease in adults using SERENE clinical trial data are underway (Study ID: NCT02065570).

This study is the first preliminary observational study that aims to evaluate the usefulness of MINI in assessing clinical response to induction therapy. In our center, due to ethical and organizational considerations, repeated endoscopies re-evaluating resolution of inflammation after EEN in children are, by default, not performed. Because of this limitation, in our study, clinical response to treatment was defined as a decrease in the PCDAI score by at least 12.5 points [11]. The PCDAI score and MINI share two variables, i.e., stool pattern and ESR. However, the interpretation of these variables (the values or weights assigned in the calculation of MINI and PCDAI) varies significantly [4,8]. Moreover, the MINI item characterizing the systemic inflammation was based on both ESR and CRP, whichever scored higher. Although the weighted Pediatric Crohn’s Disease Activity Index (wPCDAI) is better at reflecting the activity of CD, we chose not to calculate wPCDAI retrospectively. The FC assay used in our study was not able to measure concentrations higher than 1800 μg/g. In fact, many patients could have higher FC. Nonetheless, this did not affect the calculation of MINI in which FC concentrations ≥ 900 μg/g result in a maximum score of 12 points [4]. Although steroid treatment due to insufficient effects of EEN was required in eight patients (25.8%), post-EEN MINI was associated with treatment failure independently of CS. We did not aim to compare the effectiveness of induction therapy with EEN versus CS; therefore, we did not exclude children who required CS in addition to EEN. Instead, our aim was to evaluate MINI as a tool to assess the effectiveness of induction of remission. Using concomitant EEN and CS may be indicated in some pediatric CD patients [1], which is also the experience of our center. Our results are of a preliminary nature due to the limited number of patients included in the study and to the retrospective calculation of the MINI index. Individual MINI components (stool pattern, FC, ESR and C-reactive protein), however, were collected prospectively.

In our cohort, children before introducing EEN had mostly moderate to severe activity of the disease and high levels of FC. Only three children presented with pre-EEN MINI < 8 points. The only clinical feature at baseline, related to a higher risk of failing induction with EEN, was the early onset of CD (A1a—children aged under 10 years). The data comparing the effectiveness of EEN in different age groups and in children who had very early onset of CD (VEO-CD) are limited [12]. 

In our study, the pre-EEN MINI score correlated moderately with clinical activity assessment (PCDAI). It also corresponded well with clinical response to induction therapy and dropped significantly in patients with favorable treatment outcomes. A reduction of MINI by fewer than four points after EEN treatment was prognostic for its failure. In responders, a decrease in FC after EEN was observed, although the difference between pre-and post-EEN FC was smaller than previously reported in other studies [13,14]. Many reports monitoring FC during EEN in pediatric patients show that there is a significant group of children who do not normalize FC concentration after EEN treatment [13,14] as well as after CS [15], and this also occurred in our study.

To date, studies in children and in adults have shown the overall superiority of composite scores including FC, biochemical inflammatory markers and activity scores assessed with PCDAI or with the Crohn’s Disease Activity Index (CDAI) over calprotectin alone in indicating mucosal inflammation [14,16]. Cozijnsen et al. also showed that MINI is more accurate in reflecting MH than FC. In our study, post-EEN MINI, as well as post-EEN FC, discriminated responders from non-responders with high diagnostic accuracy.

A recent study in new-onset pediatric CD patients showed that, for prediction of early relapse, the response to treatment is more important than disease severity at diagnosis [17]. What is more, the FC level at the completion of EEN did not predict the time of future relapse [13]. This finding corresponds to our results. In the one-year follow-up, the post-EEN MINI score of 13 points successfully predicted CD relapse with a diagnostic sensitivity of 87.5% and specificity of 64.7%. FC did not differ in the children who experienced relapse and those with sustained response to induction therapy (*p* = 0.16). It was proved that the effect of EEN on FC diminishes soon after a normal diet is reintroduced [18]. Our results indicate that individual items included in MINI, such as the stool item and ESR were significant predictors of exacerbations, whereas FC was not. This may have significant implications for monitoring children with CD after induction of remission and suggests that MINI has more advantages than FC alone in this process.

MINI is a simple, noninvasive, clinimetric tool that allows for tight monitoring of induction treatment in pediatric CD patients. A decrease of MINI by fewer than four points after EEN is prognostic for unfavorable treatment outcomes. Overall, post-EEN MINI performed better as a prognostic factor of CD relapse than FC.

## Figures and Tables

**Table 1:** Patients’ characteristics before EEN.

Characteristic	All Patients (*n* = 31)	Responders (*n* = 24)	Non-Responders (*n* = 7)	*p*
Male sex, *n* (%)	19 (61.3)	14 (58.3)	5 (71.4)	0.5
Mean age ± SD, years	12.0 ± 3.9	12.9 ± 2.9	9.0 ± 5.6	0.1
Paris Classification				
Age:				
A1a -aged under 10 years, *n* (%)	6 (19.4)	2 (8.3)	4 (57.1)	0.004
A1b—aged 10–17 years, *n* (%)	22 (71.0)	19 (79.2)	3 (42.9)	0.063
A2—aged 17–40 years, *n* (%)	3 (9.7)	3 (12.5)	0	0.3
Location of the disease:				
L1—distal 1/3 ileum ± limited cecal disease, *n* (%)	5 (16.1)	4 (16.7)	1 (14.2)	0.9
L2—colonic, *n* (%)	11 (35.5)	7 (29.2)	4 (57.1)	0.2
L3—ileocolonic, *n* (%)	15 (48.4)	13 (54.2)	2 (28.6)	0.2
L4a—upper disease proximal to ligament of Treitz, *n* (%)	18 (58.1)	14 (58.3)	4 (57.1)	1
L4b—upper disease distal to ligament of Treitz and proximal to distal 1/3 ileum, *n* (%)	0	0	0	-
Disease behavior:				
B1—nonstricturing nonpenetrating, *n* (%)	27 (87.1)	20 (83.3)	7 (100)	0.2
B2—stricturing, *n* (%)	3 (9.7)	3 (12.5)	0	0.3
B3—penetrating, *n* (%)	0	0	0	-
B2B3—both stricturing and penetrating, *n* (%)	1 (3.2)	1 (4.2)	0	0.6
p—perianal, *n* (%)	5 (16.1)	3 (12.5)	2 (28.6)	0.3
G1—growth delay, *n* (%)	6 (19.4)	4 (16.7)	2 (28.6)	0.5

**Table 2:** Comparison of laboratory data and severity assessment pre- and post-EEN treatment in responders and non-responders.

Characteristic	pre-EEN Value in Responders	pre-EEN Value in Non-Responders	*p*	post-EEN Value in Responders	post-EEN Value in Non-Responders	*p*
Median MINI (Q1; Q3), points	19 (17; 22)	20 (12; 22)	0.6	12 (6; 14)	21 (16; 25)	0.002
Median PCDAI (Q1; Q3), points	32 (22; 36)	30 (18; 48)	1.0	1 (0; 5)	48 (15; 52)	<0.001
Median fecal calprotectin (Q1; Q3), µg/g	1800 (1368; 1800)	1042 (387; 1800)	0.1	1052 (298; 1690)	1800 (1371; 1800)	0.037
Median ESR (Q1; Q3), mm/h	26 (11; 42)	11 (8; 37)	0.3	10 (6; 19)	23 (11; 47)	0.025
Median WBC (Q1; Q3), ×10^3^/µL	8.43 (7.13; 11.92)	11.38 (4.97; 19.38)	0.4	7.01 (4.90; 8.00)	13.02 (7.40; 14.96)	0.025

Abbreviations: EEN, exclusive enteral nutrition; ESR, erythrocyte sedimentation rate; FC, fecal calprotectin; MINI, Mucosal Inflammation Noninvasive Index; PCDAI, Pediatric Crohn’s Disease Activity Index; WBC, white blood cell count.

## Data Availability

The data presented in this study are available on request from the corresponding author. The data are not publicly available due to privacy or ethical restrictions.
